# Assembly and Photocatalytic Degradation Activity of Spherical ZnO/CdSe Heterostructures on Flexible Carbon Cloth Substrates

**DOI:** 10.3390/nano12111898

**Published:** 2022-06-01

**Authors:** Xuan Chen, Jin Li

**Affiliations:** 1 Xinjiang Key Laboratory of Solid State Physics and Devices, Xinjiang University, Urumqi 830017, China; chen_xuan0916@stu.xju.edu.cn; 2 School of Physical Science and Technology, Xinjiang University, Urumqi 830017, China

**Keywords:** ZnO/CdSe–CC, heterostructure, photocatalysis

## Abstract

With the increasing water pollution, traditional treatments cannot sufficiently remove pollutants, thereby prompting the development of photocatalysts. In this study, ZnO–carbon cloth (CC) and spherical ZnO/CdSe–CC heterostructures with different CdSe loadings were synthesized using an ultrasonic-hydrothermal method on flexible CC. Z20CdSe–CC (ZnO with 20 mg CdSe loaded on CC) exhibited the best visible-light-responsive photocatalytic performance, with approximately 83.5% methylene blue reduced in 180 min. In addition, the degradation efficiency of Z20CdSe–CC was maintained at 70.9% after three cycles in relation to that of the ZnO powder. The synergistic effect of CdSe and CC not only effectively widened the light absorption range of ZnO/CdSe–CC but also further promoted the effective transfer of carriers and realized an efficient photocatalytic degradation process. Therefore, the ZnO/CdSe–CC photocatalytic material with CC as the flexible substrate exhibited high photocatalytic activity and stability in environmental remediation. This provides a design idea for the development of an efficient and flexible photocatalytic material in line with the concept of green chemistry.

## 1. Introduction

Serious pollution in freshwater resources is prevalent worldwide. As traditional adsorption, filtration, biological treatment, and other methods cannot completely remove organic and inorganic pollutants, the development of photocatalysts that can efficiently degrade pollutants has attracted the attention of researchers [1]. Zinc oxide (ZnO) is a traditional photocatalytic material with high efficiency, safety, a wide range of applications, and broad research value and development prospects [2]. Most ZnO-based photocatalysts are powder, which can cause agglomeration and loss during the photocatalytic process, thereby inhibiting their photocatalytic efficiency. Fixing ZnO photocatalysts on a flexible substrate material can address these problems [3,4,5]. Flexible substrates for photocatalysis mainly include polymer substrates, polyester fabrics, metal foils or metal meshes, and carbon materials [6,7,8,9]. However, owing to the particularity of the substrate material, most studies are limited to photocatalytic degradation using ultraviolet (UV) light, which restricts its practical application [10,11]. Therefore, ZnO photocatalysts based on flexible substrates should be modified to realize utilization in visible sunlight.

Coupling ZnO with narrow-bandgap materials is considered an effective method for improving the photocatalytic efficiency of ZnO. Cadmium selenide (CdSe) is an n-type semiconductor compound with a narrow bandgap (1.6–1.8 eV) and an electron mobility of 450–900 cm^2^·V^−1^·s^−1^, which allows easy absorption of visible light to form photogenerated carriers [12]. Thus, it is widely used in photoelectric detection, solar cells, biosensors, and photocatalysis [13]. Mahmoodi et al. [14] synthesized CdSe quantum dot–zinc oxide nanocomposites, which degraded approximately 90% BR18 dye upon UV lamp irradiation for 120 min. Li et al. [15] developed ZnO/CdSe–diethylenetriamine (DETA) nanocomposites using a two-step hydrothermal method. The hydrogen evolution rate of this nanocomposite is 11.09 mol·g^−1^·h^−1^, which is 4.32 and 123.22 times higher than those of CdSe–DETA and ZnO, respectively. Thus, the ZnO/CdSe system has great research potential owing to the combination of two semiconductor materials with excellent photoelectronic properties.

ZnO/CdSe composite structures are typically prepared using electrochemical synthesis and vapor deposition methods [16,17,18]. However, these methods have the disadvantages of harsh experimental conditions, lengthy steps, and high reaction temperatures. In addition, most of these methods use powder-type catalysts, which are easily lost during the photocatalytic process, resulting in secondary environmental pollution. Carbon cloth (CC) is a flexible material with a large specific surface area and high chemical stability. According to previous studies, the use of CC as the substrate material has a synergistic effect on the improvement of photocatalytic performance, which is beneficial to the recovery of photocatalysts [19,20]. Therefore, the growth of ZnO/CdSe on CC is a promising strategy to improve the photocatalytic performance of ZnO. However, the aforementioned design system has not been studied to date.

In this study, we proposed CC as a flexible substrate for photocatalysts and prepared ZnO/CdSe–CC layered heterostructures with the addition of different amounts of CdSe using a simple ultrasonic-hydrothermal two-step method. The phase structure, optical properties, electrochemical properties, photocatalytic efficiency, and stability of ZnO–CC and ZnO/CdSe–CC were studied. The results confirmed that the synergistic effect of CdSe and CC improved the photocatalytic efficiency of ZnO. Finally, the viability of hierarchical ZnO/CdSe–CC photocatalysts for environmental modification applications was demonstrated.

## 2. Materials and Methods

### 2.1. Materials

CC (W0S1011 CeTech Co. Ltd., Taichung, China, carbon fiber diameter of 9 μm, air permeability of 1.72, nominal basis weight of 141 g/m^2^), zinc acetate tetrahydrate (Zn(Ac)_2_·2H_2_O), zinc nitrate hexahydrate (Zn(NO_3_)_2_·6H_2_O), hexamethylenetetramine (HMTA), cadmium nitrate tetrahydrate (Cd(NO_3_)_2_·4H_2_O), and sodium selenite (Na_2_SeSO_3_) were purchased from Shanghai Aladdin Biochemical Technology Co., Ltd (Shanghai, China), hydrazine hydrate, aqueous ammonia, absolute ethanol, potassium hydroxide (KOH), and methylene blue (MB) were purchased from Tianjin Kwangfu Fine Chemical Research Institute (Tianjin, China).

### 2.2. Preparation of ZnO/CdSe–CC

#### 2.2.1. Pretreatment of CC

CC was cut into a size of 5 × 5 cm^2^ and ultrasonicated in a prepared solution of absolute ethanol and deionized water (*v*/*v*, 1:1) for 30 min. CC was then dried at 60 °C.

#### 2.2.2. Preparation of ZnO Seed Layer

The clean CC was soaked in 0.05 M Zn(CH_3_COO)_2_·2H_2_O in absolute ethanol and dried at 60 °C. This process was repeated three times. Subsequently, the dried CC was placed in a porcelain boat and thermally decomposed at 350 °C for 20 min to obtain the ZnO seed layer. This sample was named ZnO SL@CC.

#### 2.2.3. Preparation of CdSe

In 30 mL water, 4.5 mmol Cd(NO_3_)_2_·4H_2_O was dissolved, and a certain amount of aqueous ammonia was added by drops. The solution was stirred until a clear solution was achieved. Subsequently, the solution was added to 3.75 mmol Na_2_SeSO_3_ aqueous solution, and 7.5 mL hydrazine hydrate was added by drops. The mixed solution gradually turned into a dark red suspension after ultrasound treatment for 3 h and hydrothermal reaction at 70 °C for 6 h. The product was then centrifuged and washed several times. Finally, the obtained product was dried at 60 °C overnight to obtain CdSe as a dark red powder.

#### 2.2.4. Preparation of ZnO/CdSe–CC

First, 0.003 M (30 mL 100 mmol/L) Zn(NO_3_)_2_·6H_2_O aqueous solution was prepared, and a constant amount of HMTA was added by drops to the solution. After stirring for 1 h, 10, 20, and 30 mg CdSe powder were added to the solution, respectively. The suspension was homogenized by ultrasonication for 1 h and then stirred for 1 h. The prepared ZnO SL@CC and suspensions were then placed in a Teflon-sealed autoclave (Xi’an Yichuang laboratory equipment Co., Ltd, Xi’an, China) at 95 °C for 10 h. The product was washed several times and ultrasonicated for 30 s to remove soluble impurities. Subsequently, ZnO/CdSe–CC was thoroughly dried at 60 °C. Based on the amount of loaded CdSe, the samples were labeled as Z10CdSe–CC, Z20CdSe–CC, and Z30CdSe–CC. The preparation process for ZnO–CC was the same as that of ZnO/CdSe–CC, except for the absence of CdSe. For comparison in the photocatalytic experiments, ZnO powder was prepared, and the preparation conditions were the same as those of ZnO/CdSe–CC.

### 2.3. Electrochemical Testing

Electrochemical testing was performed using a three-electrode working system. Ag/AgCl electrode was used as the reference electrode, and a Pt electrode was used as the counter electrode. A small piece of ZnO/CdSe–CC was used as the working electrode. A solution of 3 M KOH was used as the electrolyte. Electrochemical impedance spectroscopy (EIS) was carried out at frequencies ranging from 100 kHz to 100 mHz.

### 2.4. Analysis of the Photocatalytic Activities

The photocatalytic performance of ZnO/CdSe–CC for the degradation of methylene blue (MB) in aqueous solutions at different times was assessed using ultraviolet-visible (UV-Vis) spectrophotometry. CC, ZnO–CC, and ZnO/CdSe–CC were cut into 5 × 5 cm^2^ samples. After weighing, the average mass of CC and ZnO–CC was 0.32 g and 0.42 g, respectively. Therefore, we speculated that the weight difference between ZnO–CC and CC was the ZnO powder loaded on CC. The 5 × 5 cm^2^ CC, ZnO–CC, ZnO/CdSe–CC heterostructure samples, and 0.1 g ZnO powder were immersed in 50 mL MB aqueous solution (10 mg/L) and stirred for 40 min in the dark to obtain an adsorption–desorption equilibrium. An 800 W xenon lamp was used to irradiate the aqueous solution at room temperature. In the adsorption–desorption equilibrium and photocatalytic process, the rotating speed of the magnetic stirring device was maintained at 150–200 rpm. The degradation efficiency was calculated using Equation (1), where C_t_ and C_0_ represent the concentrations of degraded MB at different illumination times and initial MB concentrations, respectively [21]. At the end of each cycle, the samples were washed several times and dried thoroughly before being placed in 50 mL fresh MB solution.

(1) $$\eta \left(\%\right)=\left(1-\frac{C_{t}}{C_{0}}\right)\times \;100\%$$

### 2.5. Characterization

X-ray diffraction spectroscopy (XRD) analysis was performed using a Bruker (Billerica, MA, USA) D8 Advance X-ray diffractometer to investigate the crystalline structure of the samples. The fine structures and morphologies were analyzed through a field-emission transmission electron microscope (TEM; FEI Tecnai G2 F20, Hillsboro, OR, USA) with element mapping and scanning electron microscopy (SEM; Zeiss Sigma 300, Jena, Germany). The surface states were investigated by X-ray photoelectron spectroscopy (XPS) using a Thermo Scientific (Waltham, MA, USA) ESCALAB 250Xi X. Ultraviolet-visible diffuse reflection spectrum (UV-Vis DRS) and photocatalytic performance analysis were conducted using a PerkinElmer (Waltham, MA, USA) Lambda 650 ultraviolet-visible spectrophotometer.

## 3. Results and Discussions

### 3.1. Structural Characterization

**Figure 1:**
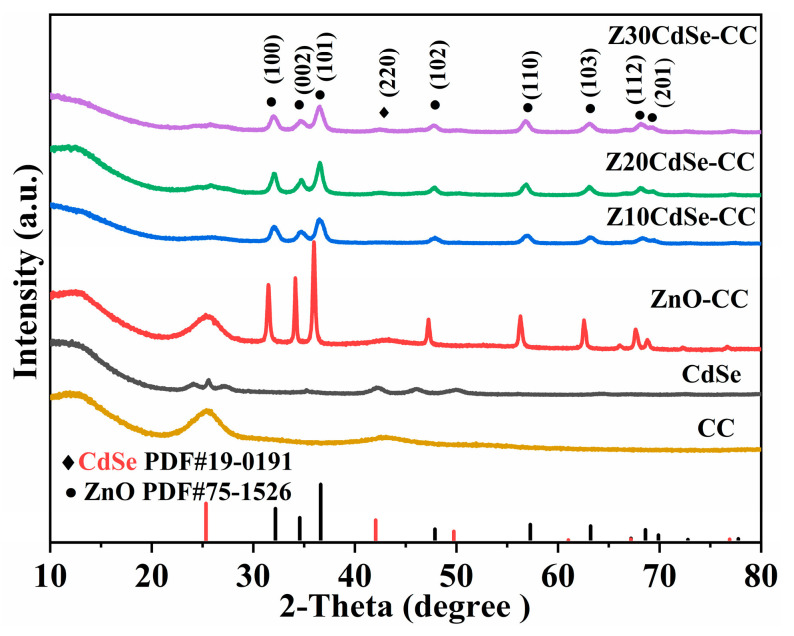
XRD patterns of CC, CdSe, ZnO–CC, Z10CdSe–CC, Z20CdSe–CC, and Z30CdSe–CC.

Figure 1 shows the XRD patterns of CC, CdSe, and ZnO/CdSe–CC heterostructures with different amounts of the CdSe composite. The XRD pattern of CdSe exhibited obvious diffraction peaks at 2θ of 25.50°, 42.10°, and 50.06°, indicating the successful formation of a CdSe cubic sphalerite structure (PDF NO. 19-0191). ZnO–CC had sharp diffraction peaks with a preferred (101) orientation. The peaks at 32.05°, 34.65°, 36.40°, 47.84°, 56.91°, 63.09°, 68.13°, and 69.26° corresponded to the hexagonal wurtzite structure of ZnO (PDF NO. 75-1526), denoted by •. Meanwhile, only one diffraction peak at 42.43° was found in the spectrum of each sample, corresponding to cubic CdSe, which is marked with ◆. Compared with the diffraction peaks of ZnO–CC, the peaks of the ZnO/CdSe–CC sample shifted to a high-angle direction, suggesting the interface effect between ZnO and CdSe. All diffraction peaks corresponded to ZnO and CdSe, implying the clean and pure phases in the synthesized samples.

According to the XRD results, the grain sizes of the (100), (002), and (101) crystal planes of the samples can be estimated using the Scherrer formula [22]:

(2) $$D=\frac{K\lambda }{\beta \cos\theta }$$

where K is the particle shape factor of 0.9, λ is the X-ray wavelength, β is the half-width of the (hkl) reflection, and θ is the Bragg angle corresponding to the (hkl) reflection. Table 1 presents the estimated results. The grain sizes of the (100), (002), and (101) planes of Z10CdSe–CC, Z20CdSe–CC, and Z30CdSe–CC were relatively similar. However, the grain size of the (002) plane of ZnO–CC was larger than that of the remaining crystal planes, implying the different morphologies of ZnO/CdSe–CC and ZnO–CC.

**Figure 2:**
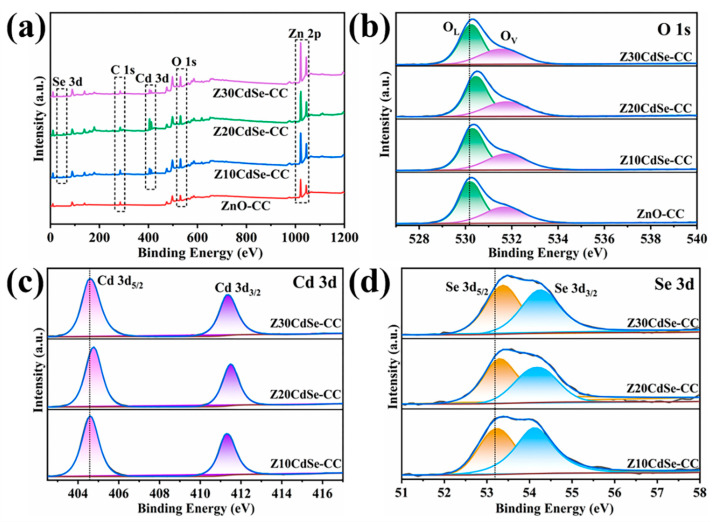
(**a**) Survey XPS spectra of each sample; high-resolution spectra of (**b**) O 1s, (**c**) Cd 3d, and (**d**) Se 3d.

To confirm the presence of CdSe and further analyze the electronic state of the related elements, ZnO–CC, Z10CdSe–CC, Z20CdSe–CC, and Z30CdSe–CC were selected for XPS characterization. All XPS data were charge-calibrated with a standard peak of 284.8 eV for C 1s. As shown in Figure 2a, Zn, O, C, Cd, and Se were observed in the survey spectra of ZnO/CdSe–CC. After fitting, the XPS spectrum of O 1s exhibited obvious shoulder peaks, which could be fitted to two peaks at 530.2 and 531.8 eV, corresponding to the lattice oxygen (O_L_) and oxygen-containing defects (O_V_) in ZnO, respectively [23]. The calculated O_L_ and O_V_ peak area ratios of ZnO–CC, Z10CdSe–CC, Z20CdSe–CC, and Z30CdSe–CC were 0.362, 1.564, 0.665, and 0.651, respectively. The increased intensity of O_V_ was ascribed to the formation of ZnO/CdSe heterostructure resulting in sensitization [24]. The two peaks located at 404.7 and 411.4 eV, as shown in Figure 2c, were attributed to Cd 3d_5/2_ and Cd 3d_3/2_, respectively, which are characteristic of typical Cd^2+^ ions [25]. In the Se 3d spectrum in Figure 2d, the peaks at the binding energies of 53.3 and 54.2 eV were associated with Se 3d_5/2_ and Se 3d_3/2_, respectively. The difference between the binding energies of Cd 3d_5/2_ and Se 3d_3/2_ was 350.5 eV, which is consistent with the theoretical value of 350.8 eV [17]. Based on a comparison of the four samples, the peak positions of all elements tended to shift toward higher binding energies. This indicated the changes in the surface electronic states around them, thereby creating a strong binding force between the two material interfaces, rather than simple physical adsorption [26]. Combined with the XRD analysis, this can be related to the generation of heterogeneous structures at the interface of ZnO and CdSe.

### 3.2. Morphological Properties

**Figure 3:**
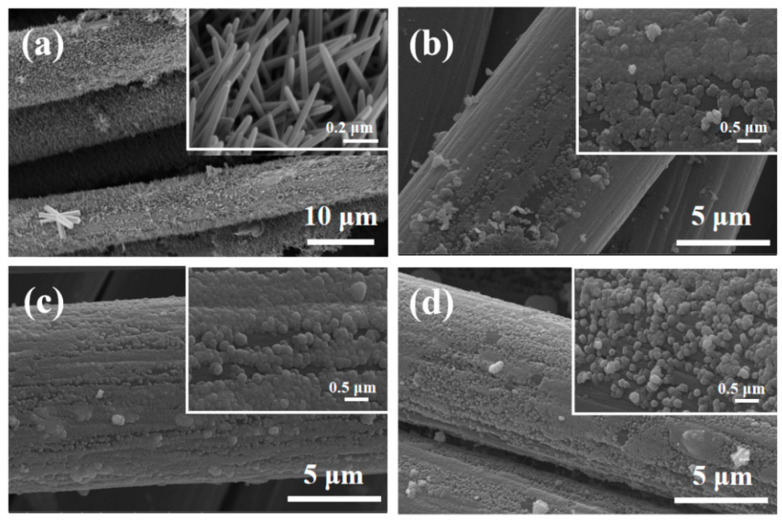
SEM of (**a**) ZnO–CC, (**b**) Z10CdSe–CC, (**c**) Z20CdSe–CC, and (**d**) Z30CdSe–CC.

The SEM images of ZnO–CC, Z10CdSe–CC, Z20CdSe–CC, and Z30CdSe–CC are shown in Figure 3. The diameter of a single carbon fiber was approximately 9 μm. For ZnO–CC, brush-like ZnO arrays were coated on the carbon fibers, as shown in Figure 3a. The diameter and length of the ZnO rods were approximately 0.2 μm and 900 nm, respectively. After compounding with different CdSe ratios, the morphology of the ZnO changed from rod-like to spherical. These microspheres were uniformly coated on the surface of the carbon fibers, which constituted the ZnO/CdSe–CC composite structure.

**Figure 4:**
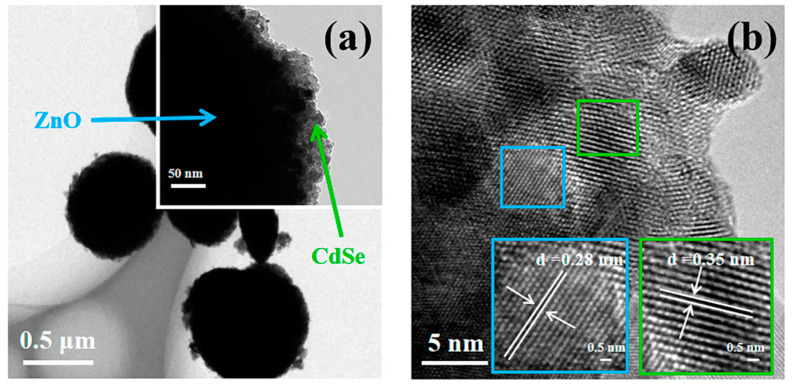
TEM (**a**) and HRTEM (**b**) of Z20CdSe–CC.

Figure 4 shows the TEM micro-area information of Z20CdSe–CC. The partially enlarged view in Figure 4a shows that the ZnO microspheres were coated with CdSe nanoparticles. Figure 4b shows the HRTEM image of Z20CdSe–CC. According to the locally enlarged view of the selected boxes, the lattice spacing was 0.28 nm, which corresponded to the (100) crystal plane of ZnO (PDF NO. 75-1526). The lattice spacing of 0.35 nm belonged to the (111) crystal plane of CdSe (PDF NO. 19-0191). These results are in perfect agreement with the XRD results.

**Figure 5:**
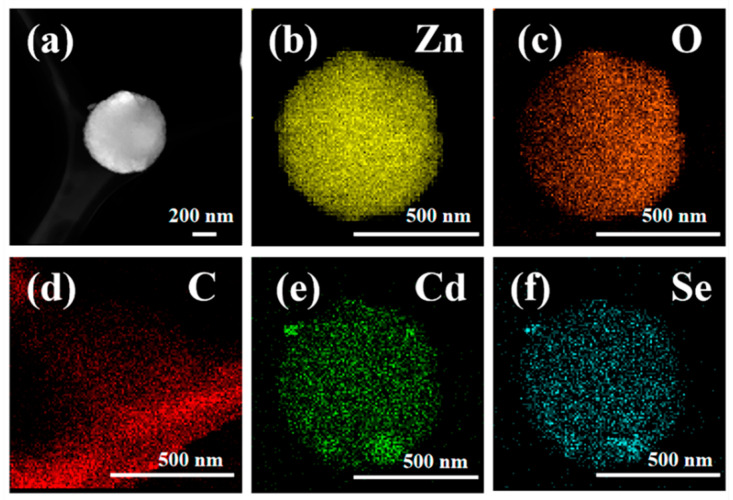
Element mapping of Z20CdSe–CC: (**a**) TEM image; (**b**) Zn, (**c**) O, (**d**) C, (**e**) Cd, and (**f**) Se.

Z20CdSe–CC was selected for further element mapping study. As shown in the diagram of different colors in Figure 5a–f, the elemental distribution was relatively uniform. The CdSe nanoparticles were coated on the surface of ZnO. The C element originated from the carbon film used in the TEM test.

**Figure 6:**
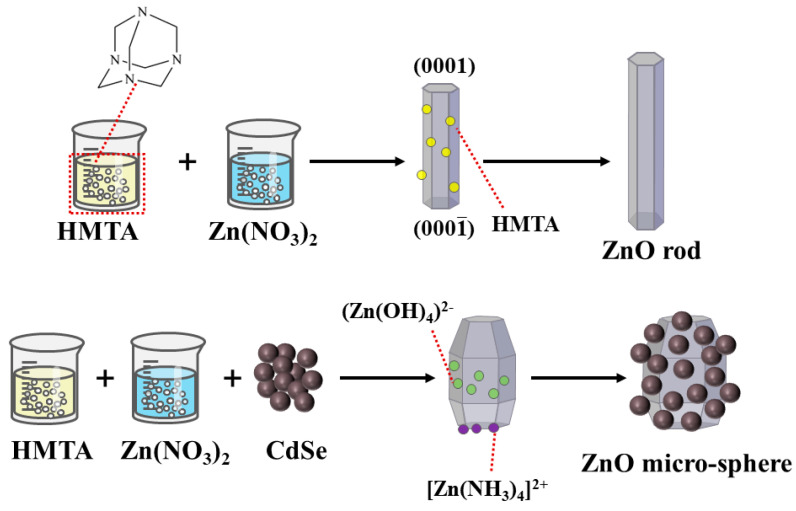
Schematic diagram of the ZnO rod and ZnO/CdSe micro−sphere growth mechanism.

Most methods for preparing ZnO rods use Zn(NO_3)2_ and HMTA, which were also used in this study [27,28]. In this study, Zn(NO_3)2_ provided Zn^2+^, and HMTA acted as a nonpolar chelating agent to link Zn^2+^ [29,30]. As shown in Figure 6, HMTA was a non-ionic cyclic tertiary amine that was hydrolyzed to produce NH_3_ and HCHO, as shown in Equation (3) [2]. The obtained NH_3_ was coordinated with Zn^2^ to ensure the stable existence of Zn^2+^, as shown in Equation (4) [29]. During the hydrothermal reaction, HMTA tended to attach to the nonpolar face, which prevented the attachment of Zn(OH)_2_ moieties and promoted the growth of the polar plane (0001) [31]. The change in the morphology from the expected rod shape to a spherical shape after the addition of CdSe may be ascribed to the induction of alkaline medium during the growth process. The growth process of a crystal is synergized by its internal structure and external environment; thus, the morphology difference of a crystal depends largely on the growth rate of the different crystal planes. As a large amount of ammonia water was added during the preparation of CdSe, residual OH^−^ caused the rapid consumption of Zn^2+^ during the hydrolysis of HMTA. It would inhibit the directional growth of the ZnO rods. Moreover, a large amount of OH^−^ caused the deposition of the growth unit (Zn(OH)_4_)^2−^ on the other crystal planes, which relatively inhibited the growth rate of the (0001) plane. The adsorption of (Zn(NH_3_)_4_)^2+^ on the negative polarity plane (000$\bar{1}$) as a chelate further slowed down the growth rate of this crystal plane [32,33]. According to the principle of minimum energy, the CdSe nanoparticles were finally agglomerated around the large ZnO particles to form microspheres with a rough surface.

(3) $${\mathrm{HMTA}+6H}_{2}O\;↔{\;4\mathrm{NH}}_{3}+6\mathrm{HCHO}$$

(4) $${\mathrm{NH}}_{3}{+H}_{2}O\;↔{\;\mathrm{NH}}_{4}{}^{+}{+\mathrm{OH}}^{-}$$

(5) $${\mathrm{Zn}}^{2+}{+4\mathrm{NH}}_{3\;}↔{\;(\mathrm{Zn}(\mathrm{NH}}_{3}{)}_{4}{)}^{2+}$$

(6) $${\mathrm{Zn}}^{2+}{+2\mathrm{OH}}^{-}\;↔{\;\mathrm{Zn}(\mathrm{OH})}_{2}$$

(7) $${\mathrm{Zn}(\mathrm{OH})}_{2}{+2\mathrm{OH}}^{-}\;↔{\;(\mathrm{Zn}(\mathrm{OH})}_{4}{)}^{2-}$$

(8) $${{\left(\mathrm{Zn}(\mathrm{OH}\right)}_{4})}^{2-}\;↔{\;\mathrm{ZnO}+4\mathrm{OH}}^{-}$$

### 3.3. Optical Properties

**Figure 7:**
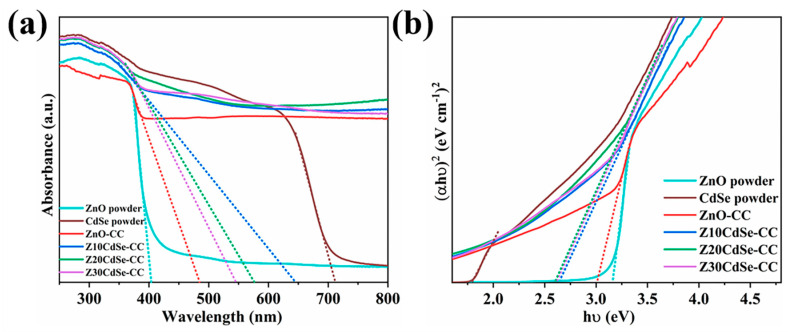
(**a**) UV-Vis DRS of ZnO, CdSe, ZnO–CC, and ZnO/CdSe−CC. (**b**) The spectra of (αhν)^2^ vs. photon energy (hν) of each sample.

To study the optical properties of the ZnO/CdSe–CC nanocomposites, the samples were tested according to the UV-Vis absorption spectrum, as shown in Figure 7a. The prepared ZnO powder exhibited the typical absorption edge of ZnO at 402 nm, which was ascribed to the wide bandgap of pure ZnO that can obtain strong absorption in the UV region. The prepared CdSe powder exhibited strong light absorption in the UV-Vis region with the absorption edge at 710 nm, indicating that the synthesized CdSe could be excited by visible light. Compared with the ZnO powder, the absorbance of the ZnO–CC and ZnO/CdSe–CC series samples in the visible region was significantly improved. This was attributed to the black CC substrate that significantly enhanced the light absorption performance of ZnO, thereby improving its photon utilization efficiency of the photocatalyst. In addition, the absorbance of the ZnO/CdSe–CC nanocomposites after compounding was further improved with the absorption edge red-shifting to the visible light region. This was due to the close combination of ZnO and CdSe semiconductors, thereby overlapping the energy bands and effectively shortening the bandgap [34]. The light absorption range of the ZnO/CdSe–CC samples was almost extended to the entire visible light band, which helped increase the concentration of the photogenerated electrons and improved their photocatalytic efficiency. The optical bandgap of each sample was roughly estimated using Tauc’s equation (αhυ)^2^ = Ahυ [35]. The estimated bandgaps are shown in Figure 7b. The E_g_ of CdSe and ZnO powders were 1.78 and 3.16 eV, respectively. Compared with the ZnO powder, the bandgap of ZnO–CC and Z20CdSe–CC were shortened to 3.00 and 2.60 eV, respectively. Combined with the change in the light absorption performance of each sample in Figure 7a and the change trend of the bandgap in Figure 7b, CdSe and CC exhibited a synergistic effect on improving photocatalytic degradation efficiency of ZnO.

### 3.4. Photoelectrocatalytic Properties

**Figure 8:**
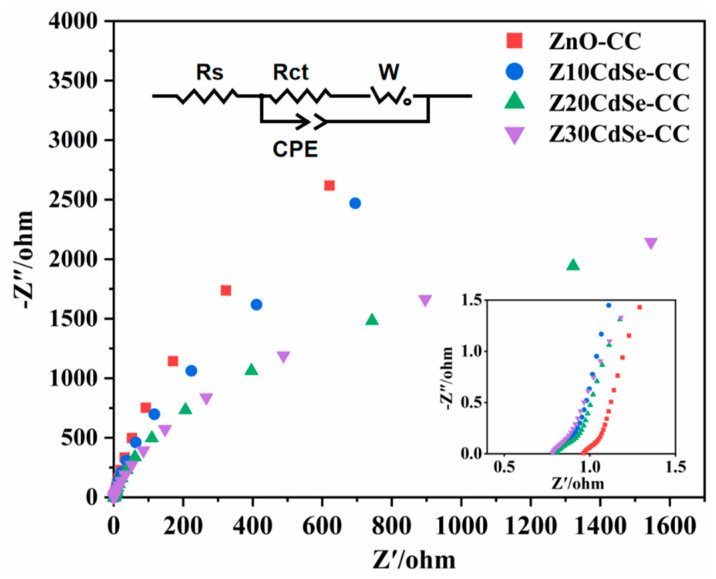
EIS Nyquist plot of ZnO−CC, Z10CdSe−CC, Z20CdSe−CC, and Z30CdSe−CC3.5. Photocatalytic Studies.

EIS is an effective method to evaluate the carrier transfer efficiency of photocatalysts, whereby the radius of the semicircular arc in each curve is related to the electrochemical reaction kinetics [36]. The carrier separation efficiencies of ZnO–CC, Z10CdSe–CC, Z20CdSe–CC, and Z30CdSe–CC were characterized, as shown in Figure 8. The radius of the arc (R_ct_) in the high-frequency region represents the ability of the ions to transfer between the electrode and electrolyte. Generally, a small R_ct_ indicates faster charge carrier transport and higher charge separation efficiency. From the partially enlarged view in Figure 8, incomplete capacitive reactance arcs can be noted, which were attributed to the low charge transfer resistance of the electrode material. The curves of these samples were fitted, and their equivalent circuit diagrams are shown in Figure 8. From the fitting results, the R_ct_ values of ZnO–CC, Z10CdSe–CC, Z20CdSe–CC, and Z30CdSe–CC were 0.2106, 0.2419, 0.2030, and 0.2066 Ω, respectively. Z20CdSe–CC had the smallest radius, which indicated effective charge transfer of the new energy band structure formed by ZnO and CdSe.

**Figure 9:**
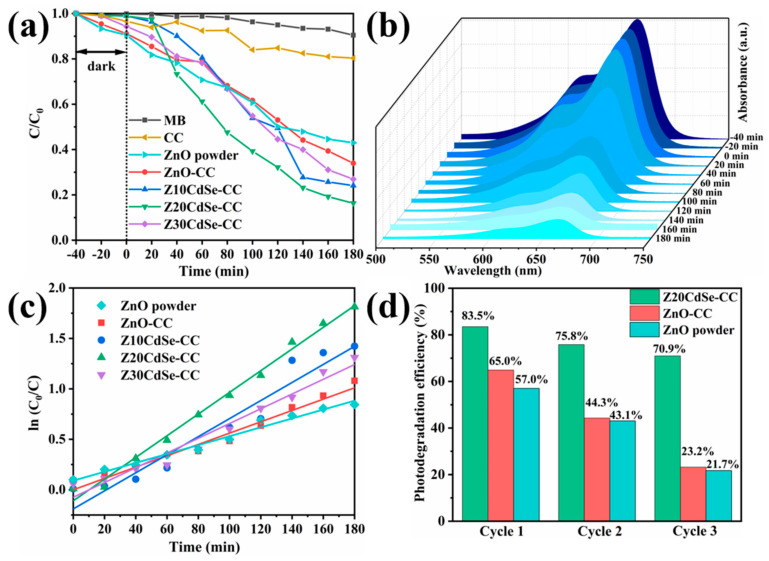
(**a**) MB degradation of each sample, (**b**) the time−dependent MB absorption spectrum of Z20CdSe−CC, (**c**) plot of the k values of all the samples, and (**d**) recycling test of ZnO, ZnO−CC, and Z20CdSe−CC.

The photocatalytic performance of the ZnO/CdSe–CC heterostructures under a xenon lamp was evaluated using 10 mg/L MB. As shown in Figure 9a, CC did not have an adsorption effect. Z20CdSe–CC had the highest degradation efficiency within 180 min, which was 83.5%, whereas ZnO powder only degraded 57.0%. However, with an increase in the CdSe loading (Z30CdSe–CC), degradation was inhibited owing to the excess CdSe covering the ZnO active sites, which decreased the catalytic activity of ZnO/CdSe–CC. The time-dependent MB spectra of the Z20CdSe–CC photocatalyst are shown in Figure 9b. The typical absorbance peak of MB at 664 nm was selected as the evaluation standard for the photocatalytic degradation. The characteristic absorption peak at 664 nm gradually weakened upon irradiation, indicating the gradual degradation of MB. Figure 9c shows the fitted curve of the first-order kinetic equation for each sample. Z20CdSe–CC had the highest degradation rate, which was 4.38 times higher than that of ZnO powder. Table 2 lists the photocatalytic parameters of the different samples in detail.

The stability of the photocatalyst is a key parameter for practical applications. The Z20CdSe–CC, ZnO–CC, and ZnO powders were selected to test the cycling stability of the degraded MB dyes for multiple cycles. As shown in Figure 9d, the degradation efficiencies of ZnO–CC and Z20CdSe–CC remained at 23.2% and 70.9%, respectively, after three consecutive uses. However, the degradation efficiency of ZnO powder without CC substrate loading was only 21.7% after three cycles. This was ascribed to the easy agglomeration and loss of the powder catalyst in the photocatalytic process, which did not occur when CC was used as the substrate. Thus, as a flexible substrate, CC has good stability and environmental protection. In addition, Z20CdSe–CC exhibited the best photocatalytic degradation efficiency and cycling stability, indicating that the synergistic effect of CC and CdSe promoted the photocatalytic performance of ZnO/CdSe–CC.

### 3.5. Photocatalytic Mechanisms

**Figure 10:**
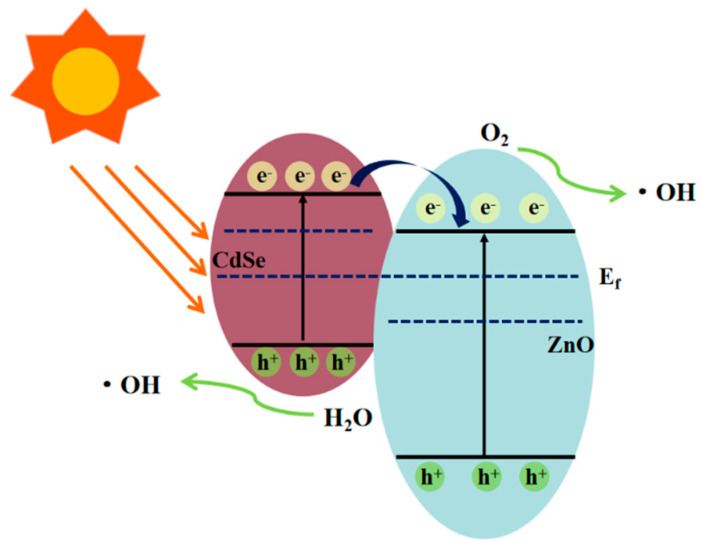
Photocatalytic mechanism of ZnO/CdSe−CC.

From the experimental results and analytical discussion, a possible photocatalytic mechanism for the ZnO/CdSe–CC heterostructure was proposed, as shown in Figure 10. According to the literature, the conduction band (CB) of CdSe is more negative than that of ZnO [37,38]. When two semiconductors combine to form a heterostructure, their Fermi levels (E_f_) are rearranged until reaching the same level. The bottom of the CB of CdSe was located in the forbidden band of ZnO, and the top of the valence bond (VB) of CdSe was located in the VB of ZnO. The energy bands of ZnO and CdSe were aligned in an interlaced manner, thereby forming a typical type-II band structure between ZnO and CdSe. Upon visible light irradiation, the narrow-bandgap material CdSe absorbed photons and produced photogenerated electrons (e^−^) and photo-production holes (h^+^). The electrons e^−^ were transferred from the CB of CdSe to the CB of ZnO, which increased the electron density on the CB of ZnO and realized the effective separation of the e^−^–h^+^ pairs. In addition, since the VB of CdSe was more negative than that of ZnO, the h^+^ in the VB of CdSe could not be transferred to the VB of ZnO, thereby increasing the concentration of h^+^ in the VB of CdSe. Further, h^+^ reacted with the H_2_O molecules adsorbed on the surface of CdSe to form highly active hydroxyl radicals (•OH) and directly oxidized with organic molecules. Simultaneously, e^−^ in the CB of ZnO reacted with the dissolved oxygen absorbed from CC in an aqueous solution to generate strong oxidizing substances such as •$O_{2}^{-}$. As CC provided abundant active sites for the close interfacial contact between ZnO and CdSe, the transfer of e^−^ was greatly enhanced. The specific process is as follows [39,40]:

(9) $$\mathrm{ZnO}/\mathrm{CdSe}+h\upsilon \;\to {\;e}_{\mathrm{CB}}^{-}{+h}_{\mathrm{VB}}^{+}$$

(10) $$e_{\mathrm{CB}}^{-}\left(\mathrm{ZnO}\right){+O}_{2}\;\to {\;•O}_{2}^{-}$$

(11) $${•O}_{2}^{-}{+H}_{2}O\;\to {\;H}_{2}O_{2}+•\mathrm{OH}$$

(12) $$h^{+}+\mathrm{MB}\;\to {\;\mathrm{CO}}_{2}{+H}_{2}O$$

## 4. Conclusions

In this study, ZnO–CC and spherical ZnO/CdSe–CC heterostructures with different CdSe loadings were synthesized using a facile ultrasonic-hydrothermal synthesis technique. The morphology changed from rod-like to spherical after the addition of CdSe, owing to the induction of the alkaline medium. In addition, Z20CdSe–CC exhibited the highest visible-light-responsive photocatalytic performance, in which approximately 83.5% of MB was reduced in 180 min. Moreover, Z20CdSe–CC exhibited the highest photocatalytic cycling stability, with its degradation efficiency being maintained at 70.9% after three cycles. The excellent photocatalytic performance of ZnO/CdSe–CC can be attributed to the following factors:

1. ZnO/CdSe–CC expanded its light absorption range, which was a prerequisite for realizing visible-light photocatalytic activity. The composite of the narrow-bandgap material CdSe significantly shortened the optical bandgap of ZnO/CdSe–CC. Moreover, the use of CC promoted the absorbance in the entire visible region. These synergistic effects enhanced the photon utilization and photocatalytic activity of ZnO/CdSe–CC.
2. The close heterojunction between ZnO and CdSe was an important factor for improving photocatalytic efficiency. Since ZnO and CdSe formed an appropriate energy band structure, the system could enhance the separation and migration of the carrier and improve the photocatalytic degradation efficiency.
3. As a flexible substrate connecting ZnO and CdSe, CC ensured the recyclability and efficient carrier separation of ZnO/CdSe. Thus, CC not only provided abundant active sites for close interfacial contact between ZnO and CdSe but also facilitated the recovery and utilization of photocatalysts.

## Figures and Tables

**Table 1:** Partial plane grain size (nm) of ZnO–CC, Z10CdSe–CC, Z20CdSe–CC, and Z30CdSe–CC.

Samples	(100)	(002)	(101)
ZnO–CC	28.72 nm	41.45 nm	30.22 nm
Z10CdSe–CC	11.80 nm	12.72 nm	12.05 nm
Z20CdSe–CC	14.04 nm	16.79 nm	15.17 nm
Z30CdSe–CC	12.38 nm	12.98 nm	13.27 nm

**Table 2:** Photocatalytic degradation parameters of different samples.

Samples	Photodegradation Efficiency (%)	*k* (min^−1^)
ZnO powder	40%	0.00245
ZnO–CC	66%	0.00562
Z10CdSe–CC	76%	0.00895
Z20CdSe–CC	84%	0.01075
Z30CdSe–CC	73%	0.00731

## Data Availability

All data included in this study are available upon request by contact with the corresponding author.
